# Bias estimation for Sigma metric calculation: arithmetic mean *versus* quadratic mean

**DOI:** 10.11613/BM.2022.030401

**Published:** 2022-08-05

**Authors:** Şerif Ercan

**Affiliations:** Department of Medical Biochemistry, Lüleburgaz State Hospital, Kırklareli, Turkey

## To the Editor:

I read with great interest the study of Keleş on the evaluation of analytical performances of clinical chemistry assays using the Six Sigma methodology (*1*). The author has computed Sigma metrics according to their laboratory performance as well as the manufacturer’s data in the reagent package inserts.

For Sigma metric calculation according to laboratory performance, the author has estimated the precision using the internal quality control data from three months, and bias by the external quality assessment (EQA) data from twelve months. Keleş has stated that the contribution of bias values to the Six Sigma budget was less than the precision. This finding has been explained by the long-term bias evaluation.

In addition, I would like to note a point for readers and the author about bias estimation.

To convert multiple bias values from the EQA surveys to a single bias value before Sigma metric calculation, the author has calculated the arithmetic mean using the following equation (Eq.):



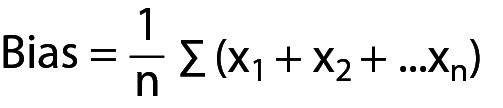



where x is the measured bias for each EQA survey and n is the number of surveys.

In my opinion, this approach is unsuitable since bias may be a negative or positive value. If one sum a negative bias with a positive bias for calculation of average bias, the resulting value will be falsely low. For example, when using a data set including - 2%, - 4%, and 6%, arithmetic mean will be computed zero while genuine bias is 4%.

Instead of the arithmetic mean, using root mean square can eliminate this shortcoming. The root mean square is also called the quadratic mean and can calculated using following equation 2.:



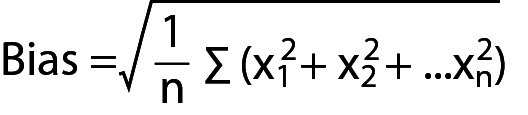



where x is the measured bias for each EQA survey and n is the number of surveys. Thus, since individual biases with negative values will be positive, the underestimation of average bias is prevented.

Apart from the EQA surveys, reducing of multiple bias values to single value has been encountered in multicenter studies on Sigma metrics in clinical laboratory. Two recent studies have estimated the pooled bias from individual site bias values obtained from method comparison studies for Sigma calculation (*2*, *3*). They have reported the pooled bias values with minus sign for some assays. This is due to calculation of the arithmetic mean for the pooled bias.

There is increasing interest in the application of Six Sigma methodology in clinical laboratory processes. For correct implementations, it is crucial to estimate properly Sigma metrics. Therefore, laboratorians should be aware on the factors that can affect the Sigma metrics. Unfortunately, no guideline exists on Sigma metric calculation and seems to be needed.
